# Embodied Conversational Agents in Clinical Psychology: A Scoping Review

**DOI:** 10.2196/jmir.6553

**Published:** 2017-05-09

**Authors:** Simon Provoost, Ho Ming Lau, Jeroen Ruwaard, Heleen Riper

**Affiliations:** ^1^ Department of Clinical, Neuro & Developmental Psychology, Section Clinical Psychology Faculty of Behavioural and Movement Sciences Vrije Universiteit Amsterdam Amsterdam Netherlands; ^2^ EMGO Institute for Health and Care Research VU University Medical Centre Amsterdam Netherlands; ^3^ GGZ inGeest Amsterdam Netherlands; ^4^ Telepsychiatry Unit Faculty of Health Science University of Southern Denmark Odense Denmark

**Keywords:** eHealth, review, embodied conversational agent, human computer interaction, clinical psychology, mental disorders, intelligent agent, health behavior

## Abstract

**Background:**

Embodied conversational agents (ECAs) are computer-generated characters that simulate key properties of human face-to-face conversation, such as verbal and nonverbal behavior. In Internet-based eHealth interventions, ECAs may be used for the delivery of automated human support factors.

**Objective:**

We aim to provide an overview of the technological and clinical possibilities, as well as the evidence base for ECA applications in clinical psychology, to inform health professionals about the activity in this field of research.

**Methods:**

Given the large variety of applied methodologies, types of applications, and scientific disciplines involved in ECA research, we conducted a systematic scoping review. Scoping reviews aim to map key concepts and types of evidence underlying an area of research, and answer less-specific questions than traditional systematic reviews. Systematic searches for ECA applications in the treatment of mood, anxiety, psychotic, autism spectrum, and substance use disorders were conducted in databases in the fields of psychology and computer science, as well as in interdisciplinary databases. Studies were included if they conveyed primary research findings on an ECA application that targeted one of the disorders. We mapped each study’s background information, how the different disorders were addressed, how ECAs and users could interact with one another, methodological aspects, and the study’s aims and outcomes.

**Results:**

This study included N=54 publications (N=49 studies). More than half of the studies (n=26) focused on autism treatment, and ECAs were used most often for social skills training (n=23). Applications ranged from simple reinforcement of social behaviors through emotional expressions to sophisticated multimodal conversational systems. Most applications (n=43) were still in the development and piloting phase, that is, not yet ready for routine practice evaluation or application. Few studies conducted controlled research into clinical effects of ECAs, such as a reduction in symptom severity.

**Conclusions:**

ECAs for mental disorders are emerging. State-of-the-art techniques, involving, for example, communication through natural language or nonverbal behavior, are increasingly being considered and adopted for psychotherapeutic interventions in ECA research with promising results. However, evidence on their clinical application remains scarce. At present, their value to clinical practice lies mostly in the experimental determination of critical human support factors. In the context of using ECAs as an adjunct to existing interventions with the aim of supporting users, important questions remain with regard to the personalization of ECAs’ interaction with users, and the optimal timing and manner of providing support. To increase the evidence base with regard to Internet interventions, we propose an additional focus on low-tech ECA solutions that can be rapidly developed, tested, and applied in routine practice.

## Introduction

### Background

Internet-based interventions can be effective in the treatment of various mental disorders compared with care-as-usual (eg, face-to-face treatment) and waiting list control groups [1]. Many interventions, especially those aimed at mood, anxiety, and substance use disorders, are based on cognitive behavioral therapy (CBT). These interventions can be unguided or guided, with guidance typically being provided by licensed health professionals or trained volunteers. Guided interventions are typically more clinically effective than unguided ones [2-4]. Their superiority is most likely explained by the interaction between the participant and the person providing guidance, and although concepts such as treatment adherence have been suggested as a working mechanism [5], that is, human support having a positive effect on adherence, and in turn on effectiveness, it remains unclear how exactly human support accounts for the difference. Thus, there is good reason to explore whether the gap between guided and unguided interventions can be bridged. In this paper we focus on a potential automated solution: Embodied conversational agents (ECAs).

### Embodied Conversational Agents

ECAs can be defined as “more or less autonomous and intelligent software entities with an embodiment used to communicate with the user” [6]. Examples of real-world ECAs are interactive characters in video games and virtual characters that assist customers in Web stores. Conceptually, ECAs consist of three components [7]. The first is an application interface that allows users to communicate with the ECA and provide it with information. These interfaces can range from Web-based questionnaires to real-time audio and video input. Second, ECAs are endowed with computer models that give them their “mental” capacities, essentially programmed knowledge used to reason over the factual “observations” derived from the interface. Such models can range from concise decision trees in which different answers on a questionnaire lead to different responses by the ECA, to machine learning–based algorithms that classify real-time video and audio input into a user’s emotional state, allowing the ECA to react empathically. Third, ECAs have an embodiment, or visual representation, which allows them to communicate with users verbally or nonverbally. Embodiments can range from virtual human characters on computer screens to robots, and communication from text messages to human communication modalities such as speech, gestures, and facial expressions. There are advantages and disadvantages to whatever implementation of the design aspects is chosen. Highly advanced ECAs, for example, those using multimodal and real-time user input such as video recordings and natural language, can be more believable than simplistic ones, but their complexity means that they require more development time, greater technological expertise, and that mistakes (eg, in interpreting semantics of natural language) become more likely. Low-tech approaches based, for example, on decision tree mechanisms or relatively simplistic graphics can be utilized to deal with these problems, but they also make for a less realistic experience. These kinds of trade-offs make finding the optimal configuration in a certain setting a nontrivial task.

### Are ECAs Ready for Clinical Practice?

Working with an existing Web- and mobile-based cognitive behavioral treatment for depression [8], our interest lies with techniques that can be applied in clinical practice. Given the many design decisions that can be made with respect to an ECA’s configuration, it is not immediately evident what an ECA should look like, and how it should behave in our context, namely as a bridge between guided and unguided interventions in clinical psychology. Paradigms exist that offer concrete design guidelines with respect to some (eg, [9]), or many (eg, [10]), of the aspects of ECA development. However, their empirical foundations generally rest on outcome measures such as “user satisfaction,” “engagement with the ECA,” or “intention to use,” and the context is not necessarily that of clinical psychology. Although such measures might be indicative of clinical effectiveness, they do not necessarily translate to the clinical outcomes we aim to improve. For example, even though users might be fully satisfied with an ECA, this does not necessarily mean that the average treatment outcome (eg, a significant reduction on a clinical measure of depression) will improve. Our chances to successfully bridge the gap between guided and unguided interventions will increase if we can determine how we can apply ECA technology with respect to improving clinical outcomes.

The interdisciplinary nature of ECA research makes almost any intervention that includes an ECA inherently complex. The UK Medical Research Council’s (MRC) framework for complex interventions [11] defines four phases through which such interventions move before being fully embedded in practice: development, piloting, evaluation, and implementation. The difference between the piloting and evaluation phase is crucial. Whereas interventions might still be subject to changes in the piloting phase, the evaluation phase is characterized by a focus on clinical outcomes and a more rigorous study design. Although routine practice sometimes evolves along different lines, as a golden rule, it is only once an intervention has successfully moved through the evaluation phase and can be considered effective and safe to use, that it becomes of practical value to psychologists.

### Scoping Review

As a first step toward designing our own ECA, we wanted to review the relevant literature in a systematic manner to find out how ECAs had previously been used in psychotherapy, and to what extent the approaches taken were supported by evidence. Initial exploration of the literature to determine a suitable review method revealed a large variety of ECA applications, study designs, and outcome measures, such that a traditional systematic review (more emphasis on hard evidence) or meta-analytic approach (requires comparable outcomes) appeared inappropriate. We therefore adopted the scoping review method [12]. Scoping reviews aim to map the key concepts underpinning a research area, as well as the main sources and types of evidence that are available [13]. Compared with traditional systematic reviews, scoping reviews address broader topics where many different study designs might be applicable, and do not emphasize quality assessment (eg, the power of the study or nature of control groups) of the included studies, as the research questions are less specific [12].

This scoping review aims to inform health professionals about the technological and clinical possibilities and evidence base for ECA applications in clinical psychology, and to provide an overview of the activity in this field of research.

## Methods

### Study Design

We adopted the Arksey and O’Malley framework for scoping reviews [12], which distinguishes five different stages: (1) identifying the research question, (2) identifying relevant studies, (3) study selection, (4) charting the data, and (5) collating, summarizing, and reporting the results. We took into account recommendations about using an iterative team approach throughout stages (1) to (4) [14,15] by having regular discussions with other team members. As theoretical underpinnings to scoping reviews, as well as transparency about the process by which results are obtained, are often lacking [16], we also made an attempt to provide clear concept definitions. Stages (1) to (4) are described in this section and stage (5) in the Results section.

### Identifying the Research Question

Given the generic features of human support in psychotherapy, techniques seen in the treatment of disorders other than depression might be applicable in our context as well. Hence, we broadened our scope to include other common mental health disorders known to be a target for e-Mental Health interventions, namely mood disorders, anxiety disorders, post-traumatic stress disorder (PTSD), psychotic disorders, eating disorders, autism spectrum disorders (ASDs), and substance-related disorders.

### Study Identification

Our generic search query was as follows:

embodied conversational agent AND mood disorder OR anxiety disorder OR psychotic disorder OR eating disorder OR autism spectrum disorder OR substance-related disorder

Our list of search terms for the ECA concept included those we observed to be most common, for example, “virtual agent,” “virtual character,” “virtual human,” or “avatar.” The terms for the mental disorders were based on those found in PubMed’s MeSH (medical subject headings) index. We searched both psychology and computer science databases, including PubMed (psychology), ScienceDirect (interdisciplinary), WebOfScience (interdisciplinary), ACM (Association for Computing Machinery) Digital Library (computer science), and SpringerLink (artificial intelligence). The detailed search strings and search procedures are described in Multimedia Appendix 1. The final search was conducted in, and included articles published up to, July 2015. References were stored in Microsoft Excel, and duplicates were removed.

**Figure 1:**
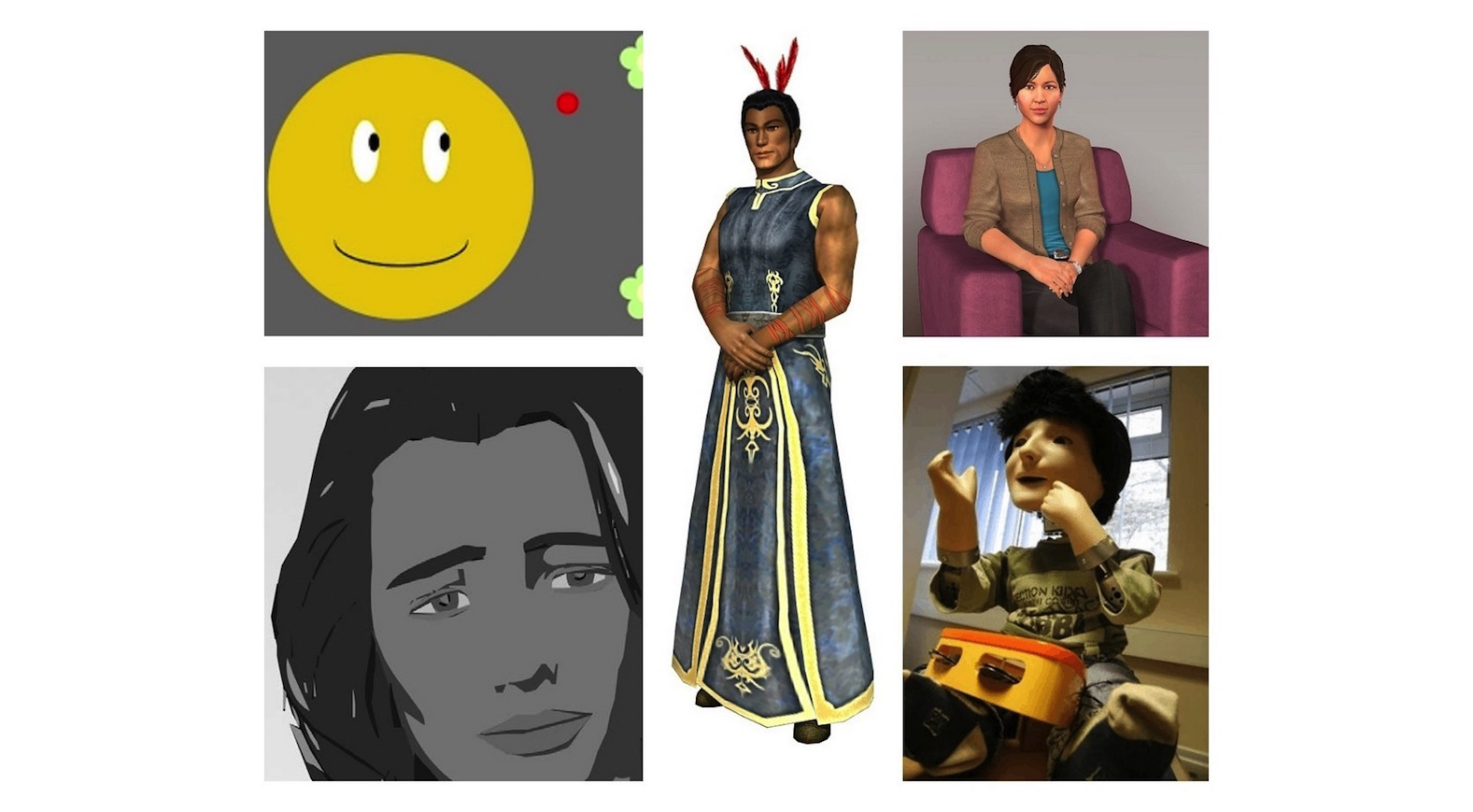
Examples of embodied conversational agent embodiments: top-left: emotional reinforcement with a smiley face; bottom-left: virtual psychiatric nurse; middle: SPARX’s (Smart, Positive, Active, Realistic, X-factor thoughts) guide character; top-right: SimSensei Kiosk virtual counselor; bottom-right: humanoid robot KASPAR.

### Study Selection

Study selection was conducted by two independent reviewers (SP & HL), who screened titles and abstracts on the sequential eligibility criteria, and then assessed the full-text versions of the remaining articles. A third reviewer (JR) was consulted in case of disagreement. We included full articles that:

(1) were written in English, (2) included an ECA in (3) an applied mental health context, (4) conveyed primary research findings, (5) targeted a mood, anxiety, psychotic, eating, autism spectrum, or substance-related disorder, and (6) described an experimental or focus group study.

Regarding criterion (2), for a software entity to be considered an ECA, it required to, first, have a virtual or physical embodiment (eg, Figure 1), second, interact with a user and, third, have a reasonable sense of agency, meaning its behavior had to be autonomous, and the software entity had to exhibit some form of reasoning. As for criterion (3), an applied mental health context implied that ECAs were used in an application that aimed to improve patient outcomes directly related to the targeted disorder, or that a reasonable argument could be made that the proposed application could eventually be used to do so.

### Charting the Data

Data extraction was conducted independently by two reviewers (SP & HL). Concepts were mapped in four categories: (1) meta-information, (2) study characteristics, (3) study methodology, and (4) ECA characteristics. Precise definitions of the concepts are listed in Multimedia Appendix 2.

During the data collection process, several concept definitions were refined. In trying to map the studies’ intended interventions and provide a taxonomy, we found that from a low level of abstraction, interventions targeted very specific behaviors or skills. Our listing grew so expansive that we considered a higher-level classification to be useful. Something similar could be said for the ECAs’ social roles, which were difficult to define precisely given the large variety in applications. In our attempt to provide a useful taxonomy, we tagged all studies with their most predominant social role and intended intervention during discussions with all reviewers present. In these discussions, definitions of the different outcome types and development phases (based on the MRC framework for complex interventions [11]) were refined as well, until each of the studies could be tagged unambiguously. The resulting definitions are also listed in Multimedia Appendix 2.

## Results

### Main Findings

**Figure 2:**
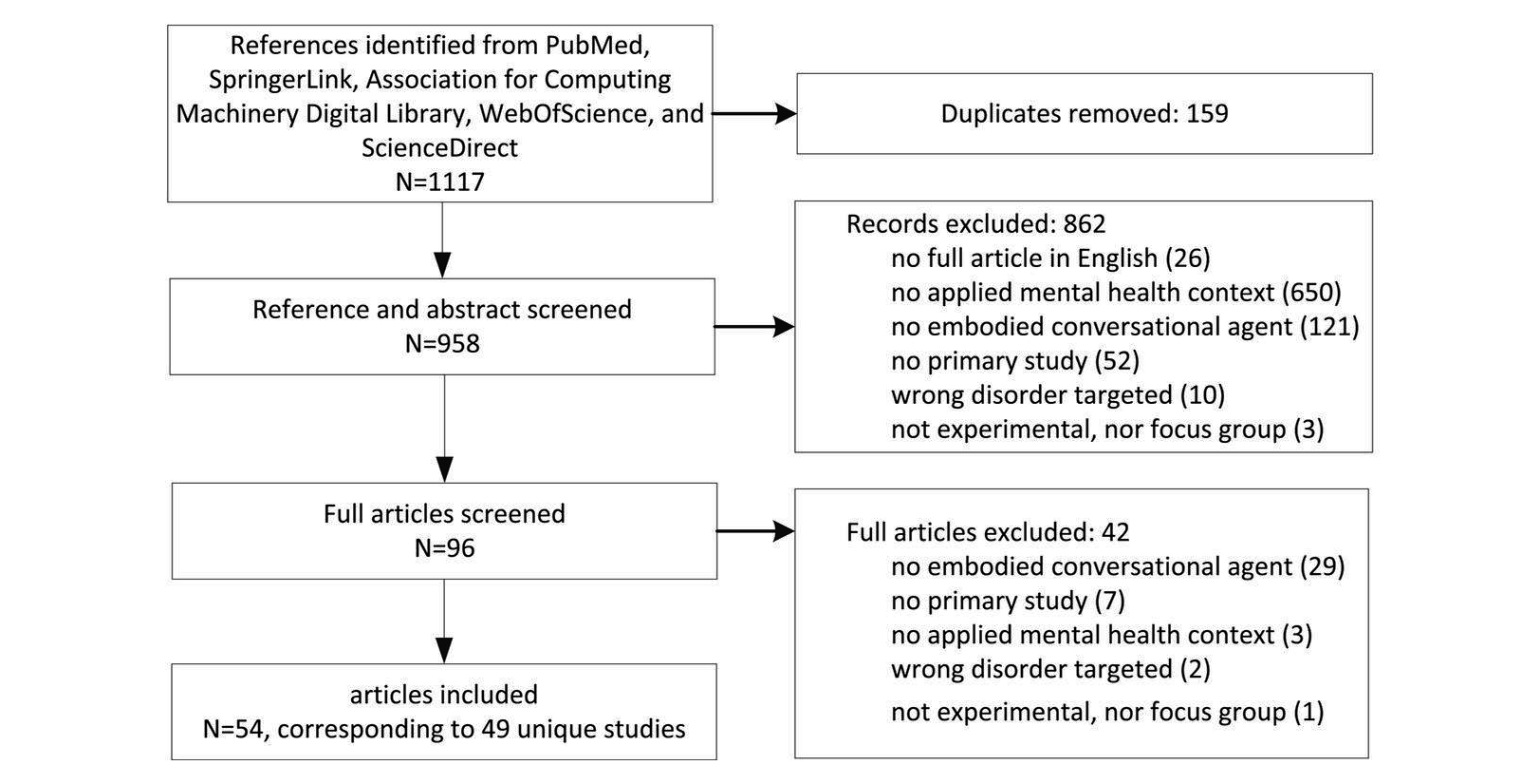
Flowchart describing study identification and selection.

**Figure 3:**
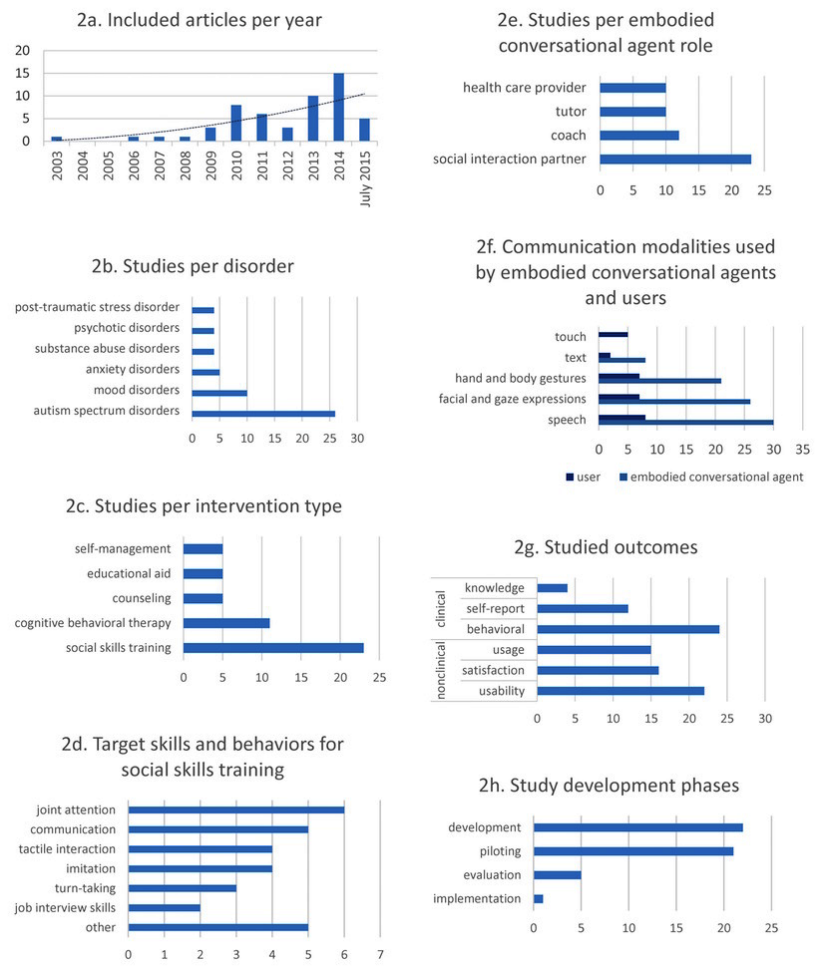
Results of the overall summative evaluation. Note that the categories were not mutually exclusive besides the intervention types and development phases.

The search identified N=1117 references. After the removal of duplicates, N=958 references remained. Next, a total of N=862 references were excluded by both reviewers after screening titles and abstracts. Of the remaining N=96 references, the reviewers did not agree about the inclusion based on screening in 78 instances, primarily because it was not entirely clear from the limited information provided by title and abstract whether the ECA inclusion criterion was satisfied (N=40). After full assessment of the 96 remaining articles, disagreement on 8 articles remained and was resolved in a discussion with the third reviewer. Finally, N=54 articles were considered eligible for full review. These 54 articles corresponded to N=49 unique studies (Figure 2). Multimedia Appendices 3 and 4, respectively, list the included studies’ intervention and ECA, and experimental design characteristics. Figure 3 depicts the most important results of the overall summative analysis and Figure 1 gives an illustration of some of the ECAs described in this review, more specifically emotional reinforcement with a smiley face [17] in the top-left, a virtual psychiatric nurse [18] in the bottom-left, SPARX (Smart, Positive, Active, Realistic, X-factor thoughts)’s guide character [19] in the middle, the SimSensei Kiosk virtual counselor [20] in the top-right, and humanoid robot KASPAR [21] in the bottom-right.

### Autism Spectrum Disorders

Over half the studies (n=26) targeted ASDs (Table 1). They either involved a form of social skills training (n=21), aimed at a variety of target behaviors or skills (Figure 3, graph d), or were presented as an educational aid (n=5) to accommodate children with autism’s special needs. Autism was the only disorder targeted with robotic applications (n=12), and most of the virtual characters appeared in serious games (n=8). Most ECAs assumed the role of a social interaction partner (n=18) or tutor (n=8), and in two studies [22,23] social interaction partners were accompanied by a coach who provided additional feedback on their performance. The relative predictability of ECA behavior compared with that of humans, the possibility to repeatedly practice certain behaviors more often than with human partners, and children with autism’s fascination for technology were important reasons to explore the use of ECA technology in autism treatment.

**Table 1:** Summative results per disorder.

Total number of studies		n=26	n=10	n=5	n=4	n=4	n=4
		ASD^a^	Depression	Anxiety	PTSD^b^	Psychotic	Substance use
**Interventions**							
	Social skills training	21	1			2	
	Educational aid	5					
	CBT^c^		4	2	1		4
	Counseling		3	3	2		
	Self-management		2		1	2	
**Platform**							
	Serious game	8	2	1	1		
	Stand-alone Software	4	2	3	1	2	
	Robotics	12					
	Virtual reality	1		1		1	
	Web based	1	6		2	1	4
**Social role**							
	Social interaction partner	18	1	3		2	
	Tutor	8		1		1	
	Coach	2	6		3	1	2
	Health care provider		5	2	1	2	2
**ECA^d^human communication modalities**							
	Speech	18	5	3	2	4	2
	Facial and gaze expressions	15	5	4	2	3	1
	Hand and body gestures	14	3	3	2	2	1
	Text	2	3	1	1		1
	Touch						
**User human communication modalities**							
	Speech	3	2	3	1	1	1
	Facial and gaze expressions	3	1	3	1		1
	Hand and body gestures	4	1	3	1		
	Text	1	1		1		
	Touch	5					
**Personalization**							
	Static user model	4					
	Dynamic user model	2	7	1	2	3	3
	Menu-based dialog	2	4		1	4	1
	Natural language dialog		2	1	2		1
**Development phase**							
	Development	13	5	2	4		1
	Piloting	11	2	3		3	2
	Evaluation	2	2			1	1
	Implementation		1				
**Outcomes**							
	Usability	11	5	2	3	3	2
	Satisfaction	3	8	1	2	4	2
	Usage	11	1	1		2	1
	Behavioral	17	2	3		3	
	Self-report	2	4	3	1	2	2
	Knowledge	4					
**Study participants**							
	Mean N (SD)	14.6 (14.2)	93.8 (108.8)	107.4 (141.0)	293 (306.7)	19.8 (11.9)	380.5 (624.8)
	Min N, max N	1, 49	8, 351	15, 351	10, 700	10, 37	35, 1317
	Clinical sample	21	7		1	4	2
	Adult sample	5	7	4	4	4	4

^a^ASD: autism spectrum disorder.

^b^PTSD: post-traumatic stress disorder.

^c^CBT: cognitive behavioral therapy.

^d^ECA: embodied conversational agent.

#### Psychotherapeutic Interventions

A first group of ASD studies targeted nonverbal communication skills. In *joint-attention skills* training, virtual characters [17,22,24-26] or a robot [27] would nonverbally cue targets by pointing or gazing at them, after which children were instructed to pay attention to the targets [17,22,24-27]. *Imitation skills* were taught by asking children to repeat nonverbal gestures made by robots [28-31]. Other applications focused on *tactile interaction* by letting children play with robots equipped with tactile sensors [31-34], *turn-taking* behavior through playing games with a virtual character [17] or robot [30], and *facial and emotion recognition* by reconstructing faces of dynamic photographs [22].

Applications focusing on verbal skills used ECAs to teach children *communication skills* such as general conversation [35,36] and antibullying strategies [37], and stimulated cooperation in multiplayer games [21,38,39]. Lastly, a *job interview training* application allowed users to practice with a virtual job interviewer [23]. It is in this group of ASD studies that we found the only three applications focusing on adults [23,35,39].

Four of the educational aid applications involved virtual tutors, targeting vocabulary [40], daily-life skills [41], general educational needs of children with ASDs [42], and comprehension of idioms [43]. One study used a humanoid robot to teach children about body postures and aid them in their sense of body consciousness [44].

#### ECA Technology

From a technological perspective, the focus in many of these studies was on letting ECAs communicate with users through human communication modalities, most notably speech (n=18), facial and gaze expressions (n=15), and hand and body gestures (n=14). A notable exception was touch (n=5), which could only be used by human users.

Few studies employed user models to personalize subsequent interactions. Static user models were mostly used to call the user by name [24,25,27,42], and only two studies employed dynamic user models to personalize subsequent interactions. In one study [26], emotion recognition was used to structure the narrative of a game, and in another [23] the interactions were structured according to the user’s performance and level of rapport with the virtual job interviewer. Only two studies allowed users to enter into a dialog with the ECA, both using menu-based dialogs [23,36].

#### Evidence

Although the most commonly studied outcomes were behavioral (n=17), and most of the studies (n=21) were conducted with clinical samples, the sample sizes were generally very small (mean 14.6 [SD 14.2]), and in many cases the behavioral outcomes were short term and restricted to experimental settings (eg, a researcher’s observation of child-robot interaction during an experiment [31]). Most studies were still in the development (n=13) and piloting (n=11) phase.

Two ASD studies had moved beyond the development and piloting phase. In the first study [23], the Web platform virtual reality (VR) job interview training was evaluated in a randomized controlled trial (N=26). Users were subjected to an interview with a job interviewer, and could ask a coach for feedback regarding their performance. Users could interact with the interviewer through a menu-based system, but they were also given the option of speaking their choices out loud to help them practice their verbal skills. The interviewer could be configured to have different personalities (from friendly and easy-going to mean and asking illegal questions), and based her responses on the user’s answers and a user model that kept track of the level of rapport between the two. The interviewer used speech output and conveyed dynamic emotional states through facial and gaze expressions. There was a significant improvement in the user’s researcher-scored interview skills in a role-played interview, as well as self-confidence, compared with a control group that received no intervention. Although this result is promising, generalizability to real-world settings and the application’s effectiveness compared with conventional job interview skills training programs remain unclear.

The second study used a computer program to improve joint attention, and emotion and face recognition in children with autism [22]. It involved exercises with dynamic realistic photographs of human faces, “coming to life” after successful completion. This intervention also included an animal avatar coach embodied by a realistic photograph that provided additional motivational reinforcement. Following a randomized controlled study (N=49), the intervention was found to be more effective in comparison to a control condition in which children used drawing software. Both children with high- and low-functioning autism improved in terms of emotion recognition and observed social skills, while children with high-functioning autism also improved in facial recognition. Similar to the other evaluation study, it remains unclear how the intervention would compare with conventional interventions targeting similar social skills.

### Depression

A total of n=10 studies targeted depression. These studies revolved around CBT interventions (n=4), counseling (n=3), self-management skills (n=2), and social skills training (n=1). Most (n=6) of the applications were Web based, and the social roles fulfilled by ECAs were a coach (n=6) or health care provider (n=5). The anonymity provided by ECAs, their availability compared with humans, their nonjudgmental nature, and the ability for people to practice social interaction in a safe environment were important reasons to explore the use of ECAs in depression treatment.

#### Psychotherapeutic Interventions

The CBT-based applications targeted *symptoms of depression* in general, with a virtual coach guiding people with depression through a Web-based intervention [45], a photograph of a clinician embodying weekly feedback in a Web-based intervention [46], and a fantasy character guiding users through a serious game [19]. In [47], requirements for the virtual agent used in [45] were determined through a focus group study.

A second group of applications explored elements of counseling with a virtual agent, more specifically the elicitation of *self-disclosure*, that is, getting people to talk about their problems, by a virtual counselor in an open-ended dialogue [20], the elicitation of self-disclosure as well as the provision of relevant information by a Web-based virtual counselor among active soldiers, war veterans, and their families [48], and *diagnosis* by a virtual therapist guiding users through a Web-based version of the Beck Depression Inventory questionnaire [49].

Self-management skills were targeted by an application that supported hospitalized patients during their *discharge procedure* [50], and by a serious game in which people with depression could practice *communicating about their health* with a virtual doctor [51].

The last study concerned the same job interview training application used in [23], this time targeting people with other psychiatric disabilities, including depression [52].

#### ECA Technology

Looking at the use of human communication modalities, the most technologically advanced developments take place in counseling interventions from studies conducted by the Institute for Creative Technologies, associated with the University of Southern California (USC-ICT). Over the years, they have developed an extensive framework that allows users to communicate with ECAs in a natural manner through verbal and nonverbal behavior. In one study [48], users could communicate by textual natural language. Using speech and synchronized nonverbal behaviors, the ECA was able to take initiative in the conversation and probe for information related to depression and PTSD. An even more advanced approach in terms of user input was taken in another study [20], in which users’ speech and nonverbal behavior was taken as input, and used to engage them in open-ended dialogues aimed at self-disclosure about psychological problems. The ECA was endowed with a set of fixed utterances and interview questions, applied back-channel behaviors (eg, saying “uhuh” and nodding while listening) and empathic responses to build rapport with the user, and used continuation prompts (eg, a new question) to keep the conversation going.

Another, technologically less advanced, approach that emphasized longer-term user modeling was taken by the Relational Agents Group of Northeastern University. During the past decade, this research group has developed a framework for so-called relational agents that apply a variety of techniques (eg, daily small-talk, empathic displays, referencing to previous interactions) to develop a long-lasting relationship with the user through menu-based dialogs over multiple interactions [50].

A last set of studies focused on modeling users’ emotional state in real time based on their interaction with the application itself, for example, based on their answers to depression questionnaires [45,49].

#### Evidence

The outcome studied the most (n=8) was user satisfaction. Although the studies focusing on more advanced technologies such as natural interaction and empathy modeling were still in the development (n=5) and piloting (n=2) phase, n=3 studies moved beyond that.

The sole study around an implementation question [19] concerned a focus group study (N=16) of the acceptability of SPARX, a gamified CBT intervention developed in New Zealand, in which players can, for example, use a staff to shoot physically manifested negative thoughts. ECAs as such are not a predominant theme in the SPARX game, but regular mention of a guide character is made. Players choose their own avatar that provides instructions throughout the game in dialogs with the user. Australian participants indicated that it was important that the guide’s gender could be customized, did not mind its foreign accent, and liked the idea of being able to socialize with it. Being a focus group study, it remains unclear whether these results would hold in an experimental setting.

The first evaluation study (N=134) used the photograph of a clinician as an embodiment to deliver automated motivational support in a computerized acceptance and commitment therapy [46]. Users could not interact with the ECA directly, and personalized support occurred on a predefined schedule through a user model based on the user’s actions in the intervention. Participants receiving automated ECA feedback were found not to be significantly less involved than those receiving real human support. Although this result was very interesting in the sense that ECA support was compared with real human support, the ECA itself made little use of state-of-the-art ECA technology, and therefore gives us little to go on in terms of ECA design. The second evaluation study concerned another randomized controlled study (N=37) using the job interview training application [52] that was also used for ASDs [23]. Again, the users’ researcher-scored interview skills in a role-played interview, as well as users’ self-confidence, improved significantly compared with a control group that received no intervention.

The development and piloting studies provided us with some initial evidence that practicing health communication with virtual health care providers in a serious game can be efficacious [51], that ECAs in a CBT-based intervention should have a coaching role, be configurable, adaptable, trustworthy, guiding rather than directive, and capable of empathic expressions without reflecting negative ones back to the user [47], that ECAs endowed with empathy are more highly valued than those without it [45,49], that people do not experience less rapport when interacting with an ECA than when interacting with a human [20], that people appreciate the anonymous nature of interacting with an ECA [48], and that people with depression experience a stronger working alliance with a virtual nurse guiding a hospital check-out procedure than do the nondepressed [50].

### Anxiety Disorders

There were N=5 studies that targeted anxiety disorders, either with CBT (n=2) or counseling (n=3) interventions. ECAs assumed the role of a social interaction partner (n=3), a health care provider (n=2), and a tutor (n=1), and most of them were implemented in stand-alone software (n=3). Reasons to use ECAs for anxiety disorders were similar to those mentioned for depression.

#### Psychotherapeutic Interventions

The counseling studies experimented with various techniques to elicit self-disclosure in counseling sessions with a virtual agent. While we already discussed one study [20] for depression, two other studies focused solely on eliciting personal information from people with anxiety in the context of finding a new roommate [53] and counseling [54]. In the CBT-based applications, virtual animals helped children to conquer performance anxiety in a serious game [55], and a virtual character evoked anxiety in a VR environment [56].

#### ECA Technology

Most innovative here are the counseling studies, again conducted by USC-ICT. Although all three ECAs are based on the same framework, those described in [53] and [54] differ from [20] in that a so-called “Wizard of Oz” paradigm was applied to control the ECAs’ verbal behavior, that is, it was controlled by the researchers. Whereas this violated our definition of agency in terms of verbal behavior, the so-called rapport agents’ nonverbal behavior was completely automated. Interpreting the phonetic aspects of a user’s speech input, as well as video recordings of his or her nonverbal behavior, they were able to display appropriate nonverbal behaviors themselves.

#### Evidence

The applications we considered were still in the development (n=2) and piloting (n=3) phase, and there was no predominant outcome measure used. Although some studies worked with large sample sizes (N=351 in [20], and N=108 in [53]), none of the studies experimented with clinical samples.

In these studies, most relevant to our purpose were the findings that people with elevated levels of social anxiety may find it easier to disclose personal information to an ECA than to a human [53], that human backstories may be more effective in this respect than (true) computer backstories [54], and that highly anxious people approached a character in a VR environment more slowly, and kept more distance, than less-anxious ones [56].

### Post-Traumatic Stress Disorder

A total of n=4 studies targeted PTSD. Besides two studies on counseling interventions that also targeted depression [20,48], one study proposed a virtual coach in a CBT-based Web-based platform [57], and one concerned a virtual guide in a serious gaming healing environment [58]. The studies involved ECAs in the role of a coach (n=3) and a health care provider (n=1).

#### Psychotherapeutic Interventions

In the first study [58], a fantasy character acted as an engaging information repository in a virtual healing environment for returning soldiers that stimulated social comradery, healing activities, and personal exploration. The other study involved a focus group of experts in trauma treatment, in which design requirements were gathered for a virtual agent supporting a Web-based exposure therapy-based application [57].

#### ECA Technology

From a technological perspective, the most interesting developments took place in the two studies we already discussed. The guide in [58] was implemented as a virtual character in a private space built in the Second Life virtual worlds platform, but the details about its design remained unclear.

#### Evidence

All studies (n=4) were still in the development phase. Even though [58] had an impressive sample size (N=700), the focus was still on usability of the healing environment itself. Some examples of suggested guidelines for the virtual coach from the focus group study (N=10) [57] were that it should acknowledge patients’ feelings, remind them of their goals when they indicate they wish to quit, be factual in complimenting, and never express negative emotion.

### Psychotic Disorders

The n=4 studies involving psychotic disorders revolved around social skills training (n=2) and self-management (n=2). Aside from the Web-based job interview training application that also targeted depression [52], two applications were implemented in stand-alone software, and one in a VR environment. Interestingly, this set of studies was the only one to consider ECAs in all four social roles. Important reasons to explore the use of ECAs in the treatment of psychotic disorders were that social skills could be practiced in a safe environment, and that ECAs can always be available to provide support or information.

#### Psychotherapeutic Interventions

Two studies applied the Relational Agent framework described in the section on depression [50], and used an ECA to host a system that provided general lifestyle support with an emphasis on promoting medication adherence for people suffering from schizophrenia over a 1-month period [18,59]. In the other study, people with schizophrenia could practice *conversational skills* with virtual characters in a VR social situation [60].

#### ECA Technology

The self-management interventions that were based on the Relational Agent framework used a similar set of techniques as in [50] to develop a long-term relationship between a virtual psychiatric nurse and the user. The conversations in [60] followed a branching tree model approach, which allowed users to communicate through a multiple choice menu. A virtual coach could help users in case they ended up in negative situations. All of the studies allowed users to interact with ECAs through menu-based dialogs.

#### Evidence

Although the studies that have not already been discussed under the various disorders were all in the piloting phase (n=3), and sample sizes were fairly small (mean 19.8 [SD 11.9]), they all studied clinical populations. Whereas usability (n=3) and satisfaction (n=4) outcomes were studied most often, with positive results, there is some initial evidence that the Relational Agent applications helped people with schizophrenia to adhere to their medication intake [18,59], and that VR social situations evoke similar negative symptoms in people with schizophrenia to what would be expected from real-world situations [60].

### Substance Use

All ECA applications (n=4) targeting substance use were Web based and included CBT elements. ECAs assumed the role of a coach (n=2) and a health care provider (n=2). The main reason to use ECAs in the context of substance use is that they are more available than supportive humans would be, thereby increasing accessibility.

#### Psychotherapeutic Interventions

In [61], a “makeover host” was used to deliver and highlight personalized content in an intervention called REALU2, targeting healthy lifestyle behavior with an emphasis on smoking cessation. Smoking cessation was also the topic of [62], which investigated the acceptability of a proposed virtual agent for an intervention based on motivational interviewing. Motivational interviewing was used in a brief intervention targeting problematic drinking behavior in [63,64].

#### ECA Technology

The makeover host in [61] delivered personalized messages based on how users interacted with the intervention, but the details of its design remained unclear. The motivational interview intervention described in [63] allowed users to interact with a virtual counselor through a menu-based system, and their facial expressions were recorded to deduce their emotional state. The combination of user input and emotional state allowed the ECA to guide the conversation and respond empathically. In [64], efforts were made to make interaction with the same system more natural by using speech rather than menu-based input.

#### Evidence

This set of studies contained one study in the evaluation phase, which had the highest number of participants (N=1317 adults with a history of smoking) out of all the studies considered. In a randomized controlled trial, the ECA intervention was found to be more effective in reducing self-reported smoking than a control condition in which participants used an intervention based on general lifestyle support. Including peer support further boosted the ECA intervention’s effectiveness. Because the ECA’s design was not described in detail, and because the intervention with the ECA was not compared with one without it, it remains unclear how the ECA itself contributed to the results. Some evidence for the importance of empathic behavior by ECAs was provided in a randomized controlled trial on the brief motivational intervention [63]. The ECA using an empathy module performed significantly better than an ECA without it on various outcome measures, but long-term effects regarding substance use remained unclear.

## Discussion

### Principal Findings

This review aimed to inform health professionals about the technological possibilities and evidence base for ECA applications in clinical psychology, and to provide an overview of the activity in this field of research. Research on the use of ECAs in psychotherapy is emerging (Figure 3, graph a), and we reviewed N=49 studies of which the majority targeted ASDs (Figure 3, graph b). A general distinction could be made between applications in which ECAs were used as an adjunct to an intervention that could also have been used independently, and applications in which the interaction between the ECA and user was central. The former were mostly CBT-based programs, educational aids, and self-management interventions, whereas the latter were mostly social interaction skills training interventions and counseling interventions. Social skills training interventions were by far the most popular for ASDs, which also made them the predominant type of intervention overall (Figure 3, graph c). As a result, ECAs in the role of a social interaction partner were the most frequent (Figure 3, graph e). The large variety in ECA applications and types of interventions (eg, Figure 3, graph d) made it a nontrivial task to provide a taxonomy of interventions and ECA social roles. Although clinical behavioral outcomes were studied most often (Figure 3, graph g), they were in many cases restricted to pre-post measurements within experiments that had relatively small sample sizes. Consequently, few studies exceeded the piloting phase (Figure 3, graph h).

#### ECA Technology

The balance in terms of the ability to communicate through human communication modalities such as gestures, expressions, and speech highly favors ECAs compared with human users (Figure 3, graph f). Nevertheless, work to shift this balance has been conducted in research on social interaction training and counseling interventions. The latter made a lot of use of technologically more advanced user modeling (eg, real-time emotional states), and innovative ways for humans to communicate with computer systems, for example, by interpreting natural language, phonetic aspects from speech input, and recorded nonverbal behavior. While ASD and counseling interventions have a more short-term focus, there has also been some activity in establishing longer-term relationships by using less technical user models, the input of which came from dialog-tree systems and indirect communication with ECAs through the intervention interface itself. Given the development phases of the studies in which these technologies were applied, the more high-tech solutions seem to be most suited for experimental research into the client-therapist relationship or screening for disorders such as depression or PTSD (eg, [20] or [48]). At present, the more low-tech approaches have been evaluated most thoroughly and, therefore, seem most promising for direct application in routine clinical practice.

#### Evidence Base

From the evaluation phase studies we learned that there is reasonable evidence that (1) ECAs can have a positive effect on user engagement and involvement, (2) they can be effective in this sense as an adjunct to already existing interventions for mental disorders, and (3) it is important for them to convey empathy when interacting with users. An important limitation of the evaluation studies we considered is the nature of the control groups. Besides the study by Kelders [46], none compared an ECA intervention to a conventional treatment used in routine practice, or compared the intervention with the ECA to the intervention without it. Additionally, the more rigorous studies either put little emphasis on the role and design of the ECAs, or targeted disorders indirectly (eg, job interviews skills). Although most of the developmental and piloting studies show promising results with respect to usability and user acceptance, we still have little hard evidence that the proposed applications are reasonable alternatives to established treatments, or that ECAs used as an adjunct to existing interventions, bridging the gap between guided and unguided interventions, make them more clinically effective.

### Future Work

In Table 1, some notable blank spots can be identified. Most obvious is the scarcity of evaluation and implementation phase studies, which requires more research with larger sample sizes, suitable control groups, and clinical populations. In this respect, the emerging nature of the field is a reassuring consideration. Two other notable blank spots are Web-based ECA applications for ASDs, and the use of ECA technology in VR applications in general. Taking into account the individual studies’ descriptions, there is also still quite some research to be done with regard to effective ECA configurations, by comparing different parameter settings in controlled studies.

#### Web-Based CBT

To revert to delivering support in Internet interventions, we know that not all people require the same amount of support, considering, for example, that people with low intrinsic motivation benefit more from human support than those who are already motivated or prefer to work on their own [5]. User models on, for example, patient motivation could contribute to an accurate timing of ECA support, for example, by providing support when motivation is low and not disturbing them when motivation is already high. Considering the working mechanisms through which human support may increase the effectiveness of Internet interventions, little work has been conducted in the area of treatment adherence, making this a pertinent target around which to focus our efforts with respect to increasing motivation.

Another important point related to extending the evidence base is illustrated by the Help4Mood project [45] and the study by Kelders [46]. The Help4Mood project attempts to integrate a Web-based CBT-based treatment with a technologically advanced ECA that communicates through speech, and that is endowed with a dynamic emotion model used to convey empathy in real time. In the study by Kelders [46], automated textual feedback in an already existing intervention was embodied by accompanying it with the photograph of a clinician. Help4Mood is technologically more challenging, but requires a long period of development and piloting. Because the ECA plays such a central role in the intervention, it is far from straightforward to simply “add” the ECA as an adjunct to an already existing and well-evaluated intervention to study its effects. Rather, the intervention would have to be built around the ECA framework, and the resulting new intervention would once again have to proceed through the development phases. This is a general issue whenever we consider adopting one of the other ECA frameworks we came across, such as the Relational Agent Group’s Litebody [65], and USC-ICT Virtual Human Toolkit [66], especially if we consider that the input used in Help4Mood is still relatively straightforward to interpret compared with human speech or nonverbal behavior.

#### Low-Tech Approach

If we want to investigate how to improve interventions that have already been set out in the field, a “low-tech” approach similar to [46] can be advocated because it (1) saves development time that can be used to design and set up larger studies, (2) forces us to think about the core attributes that can make the ECA effective, and (3) makes it easier to judge whether it is safe to use the ECA in a clinical setting with real patients. Given the sparse evidence on the clinical effectiveness of ECAs thus far, this approach and its three advantages may be just what we need to study how we can effectively use ECAs in existing Internet interventions: it will be easier to (1) conduct studies that move beyond the piloting phase, (2) identify the core attributes that make ECAs effective in Internet interventions such that ECA design can be more focused and less time-consuming, and (3) conduct experiments with clinical populations such that we can study ECAs’ effects on clinical outcomes.

### Limitations

Our definition of ECAs had three components. With respect to the embodiment and interaction capabilities, we took a liberal stance, but our requirement of agency was rather conservative (autonomous behavior and reasoning). This excluded a fair number of studies (29 during the screening of full articles) from our review that some might consider to be relevant. Regarding the criterion of autonomous behavior, we excluded quite some studies using what is often called a “Wizard of Oz” paradigm, in which the ECA’s behavior is not controlled by a software entity, but by a human operator instead. Examples are a study in which an ECA representing the hallucinated voices of people with schizophrenia spoke the transformed utterances of a therapist [67], and one in which a robot aimed at improving the mood of hospitalized children suffering from cancer was under the control of a researcher [68]. An example of a study that was excluded because the embodied characters lacked reasoning capabilities, that is, the ECA would act the same regardless of user input, was [69].

Although ECA research is almost inherently interdisciplinary, we refrained from going too deep into the technological aspects. This was because our target audience consisted of health professionals with a generally less technical background and we wanted to focus on opening up the ECA domain for them as well as providing them with an overview of the available evidence for application in routine clinical practice. For this reason, we refrained from a highly technical discussion of, for example, verbal and nonverbal ECA capabilities. However, it has to be noted that, depending on how one would like to use ECAs in future work, many more detailed questions could be investigated surrounding ECA design aspects, such as the required capabilities for, and their impact on, specific disorders or types of ECA interventions. With respect to our search strategy, we looked specifically for articles that mention ECAs. As exemplified by the sole included article on SPARX, which is actually supported by more research than reviewed here (eg, [70]), there is a possibility that we missed out on articles describing, for example, serious games or VR environments in which ECAs are used, but not specifically mentioned.

Another limitation relates to the bibliographic databases we considered. Computer science research publications are more dispersed than those of psychology research and computer science databases are less suited to systematic searches. Although our interdisciplinary approach was already broader than what is usual in psychology research, there is a possibility that we might have missed relevant research in, for example, the IEEE (Institute of Electrical and Electronics Engineers) Xplore digital library or Google Scholar. We refrained from using IEEE Xplore digital library due to practical constraints and Google Scholar because its search algorithm is often updated and personalized, which makes it difficult to replicate search results. Additionally, due to practical constraints, we did not conduct searches in the gray literature or manual searches through cross-referencing, nor did we conduct a follow-up search after the original one.

While the idea of applying ECAs in psychotherapy is far from new (eg, [71,72]), to our knowledge this is the first review to specifically consider psychotherapeutic applications of ECAs in a systematic manner. There are several areas of research that are closely related to ours. One of these is social robotics research, which often focuses on providing company to elderly people (eg, [73]) or people suffering from dementia (eg, [74]). While this area of research could be seen as an attempt to prevent the psychological consequences that might ensue from loneliness, we did not consider the focus to be on psychopathology. Robotic applications targeting autism have previously been reviewed (eg, [75]), but without the constraints implied by our ECA concept. Consequently, many of these robotic applications do not adhere to our criterion of agency, that is, they do not act autonomously or intelligently. Besides research on robotics, there is a large corpus of literature on the application of virtual agents in other highly relevant domains from which we can draw inspiration. Although this review focuses solely on psychotherapeutic applications, there seems to be little reason not to consider, for example, motivational [9], pedagogical [76], or lifestyle-support agents [77].

### Conclusions

Research into the psychotherapeutic application of ECAs is emerging. We identified 49 studies, with over half of them focusing on autism. The field is characterized by a large variety in all its aspects, for example, type of intervention, target behavior, platform, ECA embodiment, communication modalities, ECA “mental” states, and study design. While there are several studies surpassing the development and piloting phases, as might be expected in a relatively new field, evidence about the clinical effectiveness of ECA applications remains sparse. Technologically advanced ECA applications are very interesting and show promising results, but their complex nature makes it difficult for now to prove that they are effective and safe to use in clinical practice. Therefore, at present, clinical practice seems well served by an additional focus on a more low-tech approach based on the elementary principles that make ECAs effective, that can progress through the development and piloting phases at a faster pace, and that can therefore more easily be proven to be safe and effective for routine clinical practice.

## Appendix

Multimedia Appendix 1 Search strings.

Multimedia Appendix 2 Concept definitions.

Multimedia Appendix 3 Overview of the intervention aims and embodied conversational agent (ECA) characteristics.

Multimedia Appendix 4 Overview of study design characteristics by disorder.

## Abbreviations

ACM-DL: Association for Computing Machinery Digital Library
ASD: autism spectrum disorder
CBT: cognitive behavioral therapy
ECA: embodied conversational agent
MRC: UK Medical Research Council
PTSD: post-traumatic stress disorder
SPARX: Smart, Positive, Active, Realistic, X-factor thoughts
VR: virtual reality
