# Supplementary material for: Embodied Conversational Agents in Clinical Psychology: A Scoping Review
Source: J Med Internet Res. 2017 May 9;19(5):e151. doi: 10.2196/jmir.6553 (PMC5442350; doi:10.2196/jmir.6553)
Supplement: Multimedia Appendix 1 [file jmir_v19i5e151_app1.pdf]

Appendix 1. Search Strings.

PubMed (http://www.ncbi.nlm.nih.gov/pubmed/)

Search String: *("conversational agent" OR "virtual coach" OR "virtual agent" OR "embodied agent" OR avatar OR "relational agent" OR "interactive agent" OR "virtual character" OR "animated character" OR "virtual human" OR humanoid) AND ("mood disorder" OR "affective disorder" OR bipolar OR depression OR depressed OR depressive OR depression* OR depressive* OR melancholia OR anxiety OR agoraphobia OR "obsessive-compulsive disorder" OR "panic disorder" OR "phobic disorder" OR phobia OR "post-traumatic stress disorder" OR "posttraumatic stress" OR ptsd OR anxiety* OR anxious* OR phobi* OR panic* OR schizophrenia OR "psychotic disorder" OR psychoses OR psychosis OR anorexia OR binge-eating OR bulimia OR autism OR autistic OR "substance-related disorder" OR "drug dependence" OR "substance dependence" OR addiction OR "drug abuse" OR "substance abuse" OR alcoholism OR alcohol OR smoking OR tobacco OR cigarette OR nicotine OR cannabis OR marihuana OR marijuana OR "psychotherapy"[Mesh] OR "depressive disorder"[Mesh] OR "substance-related disorders"[Mesh] OR "anxiety disorders"[Mesh] OR "schizophrenia and disorders with psychotic features"[Mesh] OR "autistic disorder"[Mesh])*

WebOfScience (http://webofscience.com/)

Additional Settings: Searched on topic

Search String: *("conversational agent" OR "virtual agent" OR "embodied agent" OR "communicative agent" OR avatar OR "interface agent" OR "relational agent" OR "interactive agent" OR "believable agent" OR "game character" OR "non-playable character" OR "conversational character" OR "believable character" OR "virtual character" OR "synthetic character" OR "animated character" OR "virtual human" OR "virtual coach" OR "virtual therapist" OR "virtual assistant" OR humanoid OR ePartner) AND ("mood disorder" OR "affective disorder" OR bipolar OR depressive OR depression OR depressed OR melancholia OR anxiety OR agoraphobia OR "obsessive-compulsive disorder" OR "panic disorder" OR "phobic disorder" OR phobia OR "post-traumatic stress disorder" OR "posttraumatic stress" OR ptsd OR schizophrenia OR schizophrenic OR psychotic OR psychosis OR psychoses OR "eating disorder" OR anorexia OR bulimia OR binge-eating OR autism OR autistic OR "substance-related disorder" OR "drug dependence" OR "substance dependence" OR addiction OR "drug abuse" OR "substance abuse" OR alcoholism OR alcohol OR smoking OR tobacco OR nicotine OR cigarette OR cannabis OR marihuana OR marijuana)*

ScienceDirect (http://www.sciencedirect.com/)

Search String: *title-abstr-key(("conversational agent" OR "virtual agent" OR "embodied agent" OR "communicative agent" OR avatar OR "interface agent" OR "relational agent" OR "interactive agent" OR "believable agent" OR "game character" OR "non-playable character" OR "conversational character" OR "believable character" OR "virtual character" OR "synthetic character" OR "animated character" OR "virtual human" OR "virtual coach" OR "virtual therapist" OR "virtual assistant" OR humanoid OR ePartner) AND ("mood disorder" OR "affective disorder" OR bipolar OR depressive OR depression OR depressed OR melancholia OR anxiety OR agoraphobia OR "obsessive-compulsive disorder" OR "panic disorder" OR "phobic disorder" OR phobia OR "post-traumatic stress disorder" OR "posttraumatic stress" OR ptsd OR schizophrenia OR schizophrenic OR psychotic OR psychosis OR psychoses OR "eating disorder" OR anorexia OR bulimia OR binge-eating OR autism OR autistic OR "substance-related disorder" OR "drug dependence" OR "substance dependence" OR addiction OR "drug abuse" OR "substance abuse" OR alcoholism OR alcohol OR smoking OR tobacco OR nicotine OR cigarette OR cannabis OR marihuana OR marijuana))*

SpringerLink (http://springerlink.com)

Additional Settings: results filtered by HCI and AI

Search String: see WebOfScience

ACM Digital Library (http://dl.acm.org/)

Search String: *((Abstract:"conversational agent" or Abstract:"virtual agent" or Abstract:"embodied agent" or Abstract:"communicative agent" or Abstract:avatar or Abstract:"interface agent" or Abstract:"relational agent" or Abstract:"interactive agent" or Abstract:"believable agent" or Abstract:"game character" or Abstract:"non-playable character") and (Abstract:"mood disorder" or Abstract:"affective disorder" or Abstract:bipolar or Abstract:depressive or Abstract:depression or Abstract:depressed or Abstract:melancholia or Abstract:anxiety or Abstract:agoraphobia or Abstract:"obsessive-compulsive disorder" or Abstract:"panic disorder" or Abstract:"phobic disorder" or Abstract:phobia or Abstract:"post-traumatic stress disorder" or Abstract:"posttraumatic stress" or Abstract:ptsd or Abstract:schizophrenia or Abstract:schizophrenic or Abstract:psychotic or Abstract:psychosis or Abstract:psychoses or Abstract:"eating disorder" or Abstract:anorexia or Abstract:bulimia or Abstract:binge-eating or Abstract:autism or Abstract:autistic or Abstract:"substance-related disorder" or Abstract:"drug dependence" or Abstract:"substance dependence" or Abstract:addiction or Abstract:"drug abuse" or Abstract:"substance abuse" or Abstract:alcoholism or Abstract:alcohol or Abstract:smoking or Abstract:tobacco or Abstract:nicotine or Abstract:cigarette or Abstract:cannabis or Abstract:marihuana or Abstract:marijuana))*

((Title:"conversational character" or Title:"believable character" or Title:"virtual character" or Title:"synthetic character" or Title:"animated character" or Title:"virtual human" or Title:"virtual coach" or Title:"virtual therapist" or Title:"virtual assistant" or Title:humanoid or Title:ePartner) and (Title:"mood disorder" or Title:"affective disorder" or Title:bipolar or Title:depressive or Title:depression or Title:depressed or Title:melancholia or Title:anxiety or Title:agoraphobia or Title:"obsessive-compulsive disorder" or Title:"panic disorder" or Title:"phobic disorder" or Title:phobia or Title:"post-traumatic stress disorder" or Title:"posttraumatic stress" or Title:ptsd or Title:schizophrenia or Title:schizophrenic or Title:psychotic or Title:psychosis or Title:psychoses or Title:"eating disorder" or Title:anorexia or Title:bulimia or Title:binge-eating or Title:autism or Title:autistic or Title:"substance-related disorder" or Title:"drug dependence" or Title:"substance dependence" or Title:addiction or Title:"drug abuse" or Title:"substance abuse" or Title:alcoholism or Title:alcohol or Title:smoking or Title:tobacco or Title:nicotine or Title:cigarette or Title:cannabis or Title:marihuana or Title:marijuana))
