# Supplementary material for: Embodied Conversational Agents in Clinical Psychology: A Scoping Review
Source: J Med Internet Res. 2017 May 9;19(5):e151. doi: 10.2196/jmir.6553 (PMC5442350; doi:10.2196/jmir.6553)
Supplement: Multimedia Appendix 2 [file jmir_v19i5e151_app2.pdf]

**Appendix 2.** Concept definitions.

| Concept | Definition |
|---|---|
| **Meta-Information** | |
| Authors | The authors of the study. |
| Year of Publication | The year in which the study was published. |
| Publication Medium | The medium in which the study was published, e.g. conference proceedings, journal, or book series. |
| **Study Characteristics** | |
| Primary Institute | The research institute of the primary author. |
| Country | The country in which the primary institute is located. |
| Project Name | Name of the project or research group. |
| Targeted Disorder | The disorder that was targeted in the study. |
| Intended Intervention | |
| *Social skills training* | Improving the user's social skills |
| *CBT* | Changing cognitions or behaviors based on CBT-principles |
| *Counseling* | Getting users to communicate about their problems in a therapeutic dialogue |
| *Educational aid* | Contributing to the user's knowledge, not related to the disorder |
| *Self-management* | Stimulating the user's application of behavior change tactics to produce desired changes |
| Target Skill or Behavior | The specific skill or behavior that the intervention aimed to improve or change. |
| Number of Participants | The number of participants in the study. |
| Age Category | |
| *preschoolers* | age 0-4 |
| *children* | age 5-12 |
| *adolescents* | age 13-17 |
| *young adults* | age 18-24 |
| *adults* | age 25-64 |
| *elderly* | age 65 and older |
| Recruitment Setting | |
| *educational* | Participants were recruited through schools or universities. |
| *clinical* | Participants were recruited through health-care facilities. |
| *community* | Participants were recruited otherwise. |
| Clinical Population | Whether or not the participants were from the targeted clinical population. |
| Independent Diagnosis | Whether or not the above was verified through a reported independent diagnosis. |
| **Study Methodology** | |
| Evaluation criterion | |
| *ECA parameters* | The study evaluated particular parameter settings of the ECA (e.g. ECA gender, or a mental model). |
| *ECA* | The study evaluated whether or not the ECA itself was of additional benefit. |
| *intervention* | The study evaluated an entire intervention that included an ECA. |
| RCT | Were participants randomized in a treatment and control group? |
| Outcome Type | |
| *user satisfaction* | Whether or not users respond positively to the intervention. |
| *usability* | Whether or not users have trouble using the intervention. |
| *usage* | How often and how the intervention is used. |
| *behavioral* | Whether or not the study assesses user behavior. |
| *knowledge* | Whether or not users acquired targeted knowledge by using |

| | |
|---|---|
| | the intervention. |
| *self-report* | When users or their care-givers report on their own experience/behavior. |
| Study Aim | What the study aimed to find out. |
| Main Conclusion | The main conclusion of the study. |
| Development Phase | |
| *development* | The intervention is still subject to changes, and measures are related to usability, satisfy action, and feasibility. Measures do not yet include thorough evaluation based on relevant clinical outcomes. These types of studies describe interesting new developments that require further evaluation. |
| *piloting* | The intervention is near completion, and relevant patient outcomes are taken into account in the evaluation. Usability, satisfaction, and feasibility outcomes can go hand-in-hand with patient outcomes. Evidence is not yet significant enough to give clinicians enough confidence to apply it in practice. |
| *evaluation* | The evaluation revolves primarily around the intervention's effect on clinical outcomes. Sample sizes are typically larger, and methodology is more rigorous. These interventions have been evaluated to the extent that clinicians could consider their practical application. |
| *implementation* | The intervention has already gone through the evaluation phase, and has been used in practice for some time. |
| **ECA Characteristics** | |
| Platform | The platform in which the ECA was embodied. |
| *serious game* | A game with a primary purpose other than entertainment |
| *stand-alone software* | Platforms that do not belong to any of the other categories |
| *robotics* | Platforms with a physical embodiment rather than a virtual one |
| *virtual reality* | Truly immersive (more so than just a computer screen) applications |
| *web-based* | Applications that run in a web-browser |
| Platform Specification | A further specification of the platform if applicable. |
| Personification | Who or what the ECA embodiment personifies. |
| Personification Specification | A further specification of the personification if applicable. |
| Social Role | |
| *Social interaction partner* | To engage in an interaction with the user to improve specific social skills |
| *Tutor* | To teach something to the user |
| *Coach* | To motivate and engage the user |
| *Health-care provider* | To simulate the behavior of a health-care provider |
| Purpose | What it is that the ECA does in the application. |
| Dialog | Whether or not users could enter into a dialog with the ECA. |
| Dialog Specification | How users could enter into the dialog. |
| Human-ECA Interaction | How users could communicate with the ECA. |
| Human Communication Modalities Used | Whether this involved human communication modalities. |
| Communication Modalities | If so, which communication modalities. |
| ECA-Human Interaction | How the ECA communicated with the user. |
| Human Communication Modalities Used | Whether this involved human communication modalities. |
| Communication Modalities | If so, which communication modalities |
| User Model | Whether the ECA kept track of information on the user to personalize the interactions. |
| *static* | the model was based on information entered before the interactions (e.g. participant age) |
| *dynamic* | the model was updated throughout the interactions to keep track of a user's changing states (e.g. emotional state) |
