# Supplementary material for: Embodied Conversational Agents in Clinical Psychology: A Scoping Review
Source: J Med Internet Res. 2017 May 9;19(5):e151. doi: 10.2196/jmir.6553 (PMC5442350; doi:10.2196/jmir.6553)
Supplement: Multimedia Appendix 3 [file jmir_v19i5e151_app3.pdf]

Appendix 3. Overview of the intervention aims and ECA characteristics.

Disorder/Author	Intervention	Intervention targets	Platform	ECA Embodiment	ECA Social Role
**Autism**					
Agarwal (2013)	social skills training	turn-taking, joint attention	game	fantasy, smiley	social interaction partner
Alcorn (2011)	social skills training	joint attention	game	human	social interaction partner
Amirabdollahian (2011)	social skills training	tactile interaction	robotics	humanoid	social interaction partner
Bamasak (2013)	educational aid	cognitive, social, and self-care skills	game	human	tutor
Bekele (2013)<br>Bekele (2014)	social skills training	joint attention	robotics	humanoid	social interaction partner
Bernardini (2012)<br>Bernardini (2014)	social skills training	joint attention	game	human	social interaction partner
Boccanfuso (2010)	social skills training	turn-taking, imitation	robotics	toy-like	social interaction partner
Chen (2010)	social skills training	joint attention	game	human	social interaction partner
Cole (2003)	educational aid	vocabulary	stand-alone	human	tutor
Costa (2015)	social skills training	tactile interaction, body consciousness	robotics	humanoid	social interaction partner
Dickerson (2013)	social skills training	tactile interaction	robotics	humanoid	social interaction partner
Fujimoto (2010)<br>Fujimoto (2011)	social skills training	imitation	robotics	humanoid	social interaction partner
Hopkins (2011)	social skills training	joint attention, facial & emotion rec	game	photograph	social interaction partner, coach
Jordan (2013)	social skills training	communication, turn-taking	robotics	toy-like	social interaction partner
Kim (2010)	social skills training	positive play	robotics	toy-like	social interaction partner
Konstantinidis (2009)	educational aid	special needs	stand-alone	human	tutor
Lahiri (2011)	social skills training	communication	virtual reality	human	social interaction partner
Milne (2009)	social skills training	communication	stand-alone	human	tutor
Palestra (2014)	educational aid	body consciousness	robotics	humanoid	tutor
Ribeiro (2014)	social skills training	communication	game	human	tutor
Robins (2014)	social skills training	tactile interaction, imitation	robotics	humanoid	social interaction partner
Shoukry (2015)	educational aid	learning idioms	game	human	tutor
Smith (2014b)	social skills training	job interview skills	web-based	human	social interaction partner, coach
Tanaka (2015)	social skills training	communication	stand-alone	human	tutor
Wainer (2014a)<br>Wainer (2014b)	social skills training	cooperation	robotics	humanoid	social interaction partner
Warren (2014)	social skills training	imitation	robotics	humanoid	social interaction partner

**Depression**					
Bickmore (2010a)	self-management	hospital discharge	stand-alone	human	health-care provider
Cheek (2014)	CBT	symptoms of depression	game	fantasy	coach
Kelders (2015)	CBT	symptoms of depression	web-based	photograph	health-care provider
Martínez-Miranda (2014)	CBT	symptoms of depression	web-based	human	coach
Pagliari (2012)	CBT	symptoms of depression	web-based	unknown	coach
Pinto (2013)<br>Pinto (2015)	self-management	health communication	game	human	health-care provider, coach
Pontier (2008)	counseling	clinical assessment	web-based	human	health-care provider
DeVault (2014)[a]	counseling	self-disclosure	stand-alone	human	health-care provider
Swartout (2013)[b]	counseling	screening	web-based	human	coach
Smith (2014a)[c]	social skills training	job interview skills	web-based	human	social interaction partner, coach
**Anxiety**					
Kang (2010)	counseling	self-disclosure	stand-alone	human	social interaction partner
Kang (2012)	counseling	self-disclosure	stand-alone	human	health-care provider
Rinck (2010)	CBT	exposure to social situations	virtual reality	human	social interaction partner
Schmidt (2013)	CBT	performance anxiety	game	animal	social interaction partner, tutor
**PTSD**					
Morie (2009)	self-management	symptoms of PTSD	game	fantasy	coach
Tielman (2014)	CBT	memory restructuring	web-based	unknown	coach
**Schizophrenia**					
Bickmore (2010b)	self-management	medication adherence	stand-alone	human	health-care provider
Ku (2007)	social skills training	conversation skills	virtual reality	human	social interaction partner, tutor
Puskar (2011)	self-management	medication adherence	stand-alone	human	health-care provider
**Substance Abuse**					
An (2013)	CBT	smoking cessation	web-based	human	coach
Grolleman (2006)	CBT	smoking cessation	web-based	human	coach
Lisetti (2013)	CBT	alcohol dependency	web-based	human	health-care provider
Yasavur (2014)	CBT	alcohol dependency	web-based	human	health-care provider

Note. CBT = Cognitive Behavioral Therapy; ECA = Embodied Conversational Agent; PTSD = Post-Traumatic Stress Disorder

a: also targeted anxiety and PTSD, **b**: also targeted PTSD, **c**: also targeted schizophrenia
