# Supplementary material for: Embodied Conversational Agents in Clinical Psychology: A Scoping Review
Source: J Med Internet Res. 2017 May 9;19(5):e151. doi: 10.2196/jmir.6553 (PMC5442350; doi:10.2196/jmir.6553)
Supplement: Multimedia Appendix 4 [file jmir_v19i5e151_app4.pdf]

Appendix 4. Overview of study design characteristics by disorder.

Disorder/Author	N	Target Group	Clinical Sample	Outcomes	Development Phase
**Autism**					
Agarwal (2013)	4	children, adolescents	yes	usability	development
Alcorn (2011)	32	children, adolescents	yes	usability, behavioral	development
Amirabdollahian (2011)	-	preschoolers	yes	usage	development
Bamasak (2013)	10	children, adolescents	yes	usability	development
Bekele (2013)<br>Bekele (2014)	12	preschooler	yes	usability, usage, behavioral	piloting
Bernardini (2012)<br>Bernardini (2014)	19	preschoolers, children, adolescents	yes	behavioral, usage	piloting
Boccanfuso (2010)	-	children	no	usage	development
Chen (2010)	-	children	yes	usage	development
Cole (2003)	6	preschoolers, children	yes	knowledge, behavioral	piloting
Costa (2015)	8	children	yes	behavioral, knowledge, usage	piloting
Dickerson (2013)	1	children	yes	behavioral	piloting
Fujimoto (2010)<br>Fujimoto (2011)	4	children	yes	behavioral, usage	development
Hopkins (2011)	49	children, adolescents	yes	behavioral, self-report	evaluation
Jordan (2013)	5	young adults	yes	behavioral, usage	piloting
Kim (2010)	3	preschooler	yes	behavioral	development
Konstantinidis (2009)	13	adults	no	self-report, usability	development
Lahiri (2011)	4	adolescents	yes	behavioral	development
Milne (2009)	7	children, adolescents	unknown	knowledge, usability	development
Palestra (2014)	10	young adults	no	satisfaction, usability	development
Ribeiro (2014)	4	children	yes	behavioral	piloting
Robins (2014)	36	preschoolers, children	no	behavioral, usability	piloting
Shoukry (2015)	12	children, adolescents	yes	usability, knowledge, usage	development
Smith (2014b)	26	adults	yes	usability, usage, satisfaction, behavioral	evaluation
Tanaka (2015)	49	young adults	no	behavioral, usability, satisfaction	piloting
Wainer (2014a)<br>Wainer (2014b)	6	unknown	yes	behavioral	piloting
Warren (2014)	16	preschoolers	yes	behavioral, usage	piloting
**Depression**					
Bickmore (2010a)	131	young adults, adults	yes	satisfaction, usability, self-report	piloting
Cheek (2014)	16	adolescents	yes	satisfaction	implementation
Kelders (2015)	134	adults	yes	behavioral, self-report	evaluation
Martínez-Miranda (2014)	8	young adults, adults	yes	satisfaction	development
Pagliari (2012)	-	unknown	yes	unknown	development
Pinto (2013)<br>Pinto (2015)	28	young adults	yes	self-report, satisfaction	piloting

Pontier (2008)	28	unknown	no	satisfaction	development
DeVault (2014)[a]	351	adults	no	satisfaction, usability, self-report	development
Swartout (2013)[b]	111	adults	no	usability, satisfaction	development
Smith (2014a)[c]	37	adults	yes	usability, usage, satisfaction, behavioral	evaluation
**Anxiety**					
Kang (2010)	108	adults	no	behavioral	piloting
Kang (2012)	40	adults	no	behavioral	piloting
Rinck (2010)	23	young adults	no	self-report, behavioral	piloting
Schmidt (2013)	15	children	no	usability, self-report, usage	development
**PTSD**					
Morie (2009)	700	adults	yes	usability	development
Tielman (2014)	10	adults	no	unknown	development
**Schizophrenia**					
Bickmore (2010b)	15	young adults, adults	yes	satisfaction, usage, self-report	piloting
Ku (2007)	10	adults	yes	satisfaction, usability, behavioral, self-report	piloting
Puskar (2011)	17	young adults, adults	yes	behavioral, satisfaction, usability	piloting
**Substance Abuse**					
An (2013)	1317	young adults, adults	yes	self-report	evaluation
Grolleman (2006)	35	young adults	yes	unknown	development
Lisetti (2013)	81	young adults	no	usability, satisfaction, self-report	piloting
Yasavur (2014)	89	young adults	no	usability, satisfaction, usage	piloting

Note. ‘-‘ indicates that sample sizes were not reported.

a: also targeted anxiety and PTSD, **b**: also targeted PTSD, **c**: also targeted schizophrenia
